# Massive Arterial Cerebral Thrombosis in a 59-year old Female Patient With Severe COVID-19

**DOI:** 10.7759/cureus.15553

**Published:** 2021-06-09

**Authors:** Osama B Albasheer, Haneen A Almutairi, Fayez M Almalki, Hani M Malaka

**Affiliations:** 1 Family and Community Medicine, Jazan University, Jazan, SAU; 2 Internal Medicine, East Jeddah General Hospital, Jeddah, SAU; 3 Neurology, East Jeddah General Hospital, Jeddah, SAU; 4 Radiology, East Jeddah General Hospital, Jeddah, SAU

**Keywords:** covid-19, arterialthrombosis, coagulopathy, global pandemic, inflammation

## Abstract

Hypercoagulable states characterized by micro- and macro-vascular thrombotic angiopathy have been observed in COVID-19 patients. Although venous thrombotic events have been well described, data on arterial thrombosis (AT) is still insubstantial. We present a case of COVID-19 complicated with massive arterial cerebral thrombosis. Our case is a 59-year old female with history of hypertension who presented to the outpatient clinic in East Jeddah Hospital, Saudi Arabia, with sore throat, cough and arthralgia for two days. She was confirmed to be COVID-19 positive and started on azithromycin and supportive home care. Her condition worsened and she presented nine days later with drowsiness and generalized weakness. At the hospital, she was diagnosed with severe COVID-19 and was started on prophylaxis enoxaparin. She showed progressive worsening of mental function. Her CT brain showed diffuse extensive arterial cerebral thrombosis. She remained unresponsive and showed an abnormal breathing pattern on mechanical ventilation. She died on day 4 after admission.

## Introduction

The novel Severe Acute Respiratory Syndrome Coronavirus 2 (SARS-CoV-2) was declared by WHO as a public health emergency of global concern [1]. A recent study postulated that severe cases of COVID-19 can cause abnormal coagulation, which was associated with increase mortality [2]. Pulmonary thromboembolism and small arterial vessel thrombosis were well reported in the literature [3,4]. Only small series are available on systemic arterial thromboembolic disease of the larger vessels [5].

## Case presentation

A 59-year old female with a history of hypertension presented to the outpatient clinic in East Jeddah Hospital, Saudi Arabia, with sore throat, cough and arthralgia for 2 days. A nasopharyngeal swab for COVID-19 testing was performed as per the protocol during the current COVID-19 pandemic. The swab returned positive for COVID-19 and the patient was started on azithromycin (500 mg orally for 3 days) and paracetamol (1 g BID orally). Nine days later, the patient presented to the emergency department with drowsiness, loss of appetite and decreased activities. Patient was admitted and was initiated on prophylaxis enoxaparin 30 mg subcutaneously q12hr.

**Figure 1:**
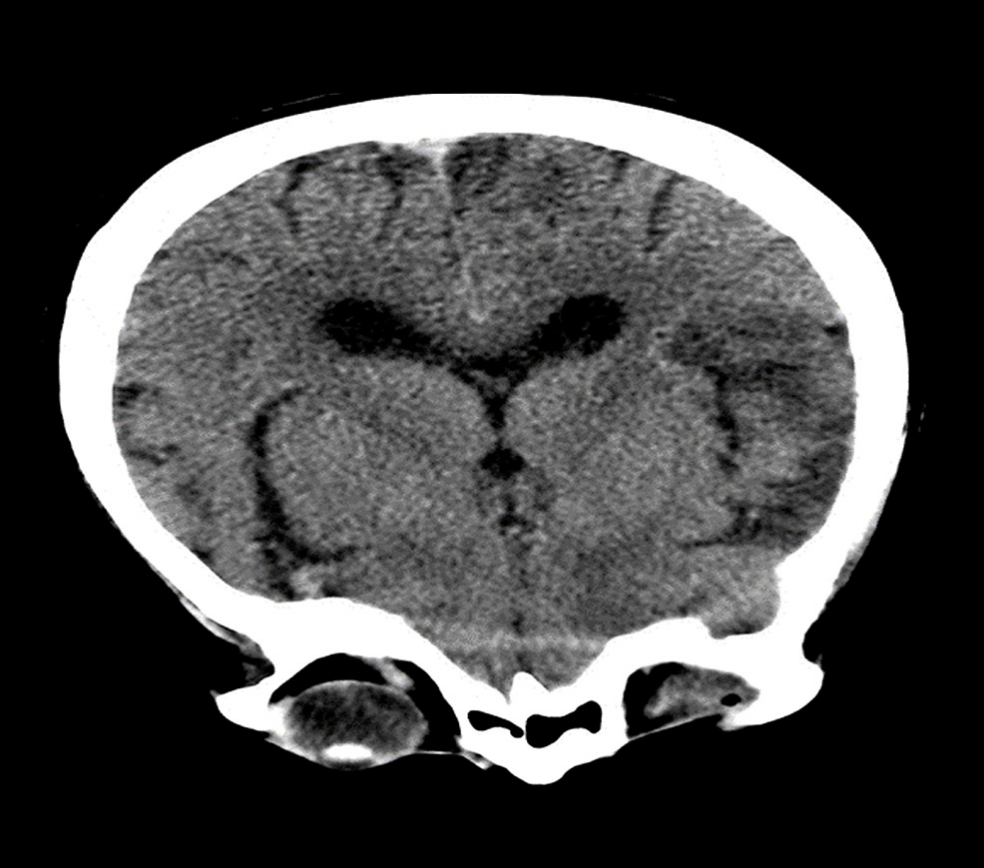
CT brain on the first day of admission Brainstem and cerebellum without evidence of focal lesions. Ventricles are normal size. Midline is straight.

**Figure 2:**
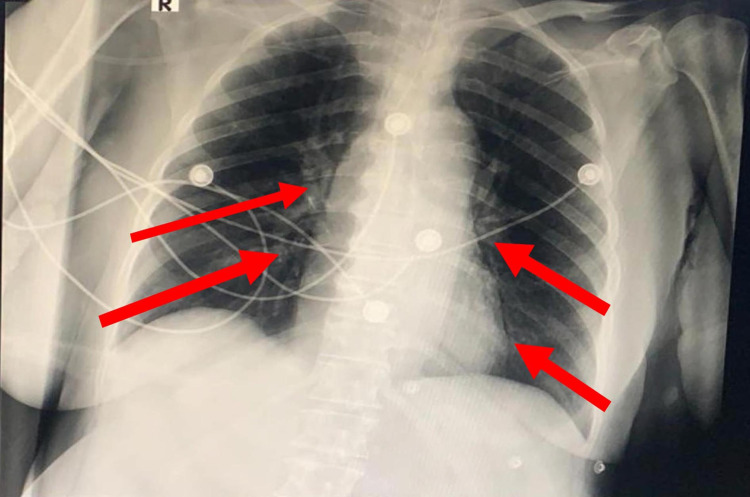
Chest x-ray on the first day of admission Ground glass opacities (red arrows) are seen involving the central and peripheral zones bilaterally

**Figure 3:**
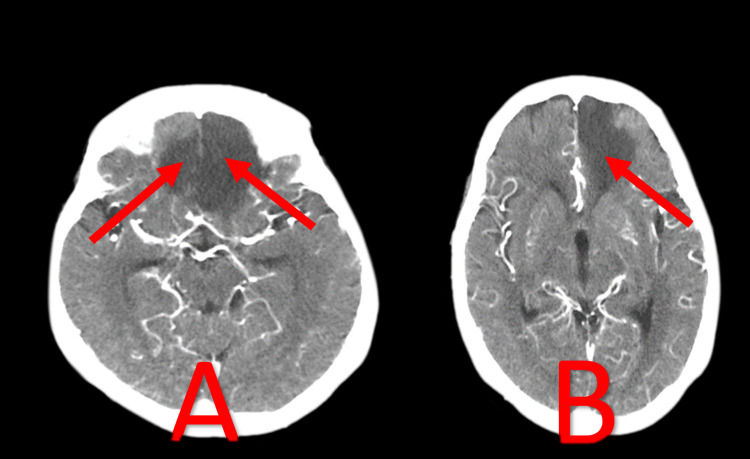
CT brain on the 2nd day post admission Bilateral frontal ischemia. Image A showed diffuse hypo-dense lesions involving the parasagittal part of the right and left frontal lobes (red arrows). Image B showed hypo-dense lesions involving the parasagittal part of the left frontal lobe

**Figure 4:**
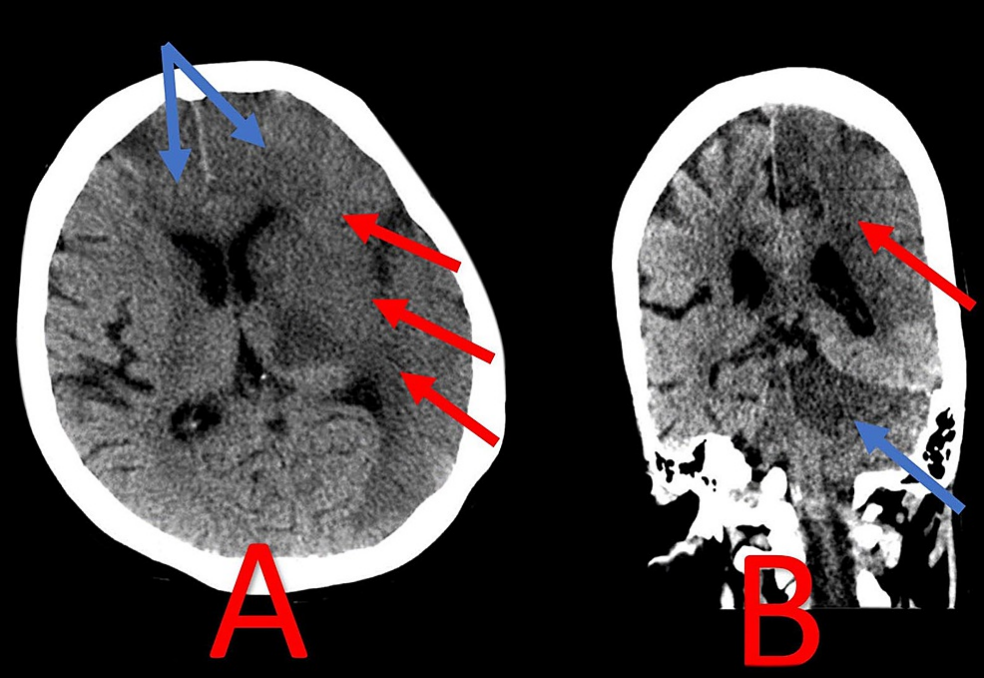
CT brain extensive involvement of right frontal lobe and cerebellum Image A showed hypo-dense lesions (blue arrows), involving the parasagittal area of the right and left frontal lobes and left temporal lobe (red arrows). Image B showed hypo-dense lesions involving the parasagittal area of the right frontal lobe (blue arrows) and  most of the right cerebellum(red arrows).

**Figure 5:**
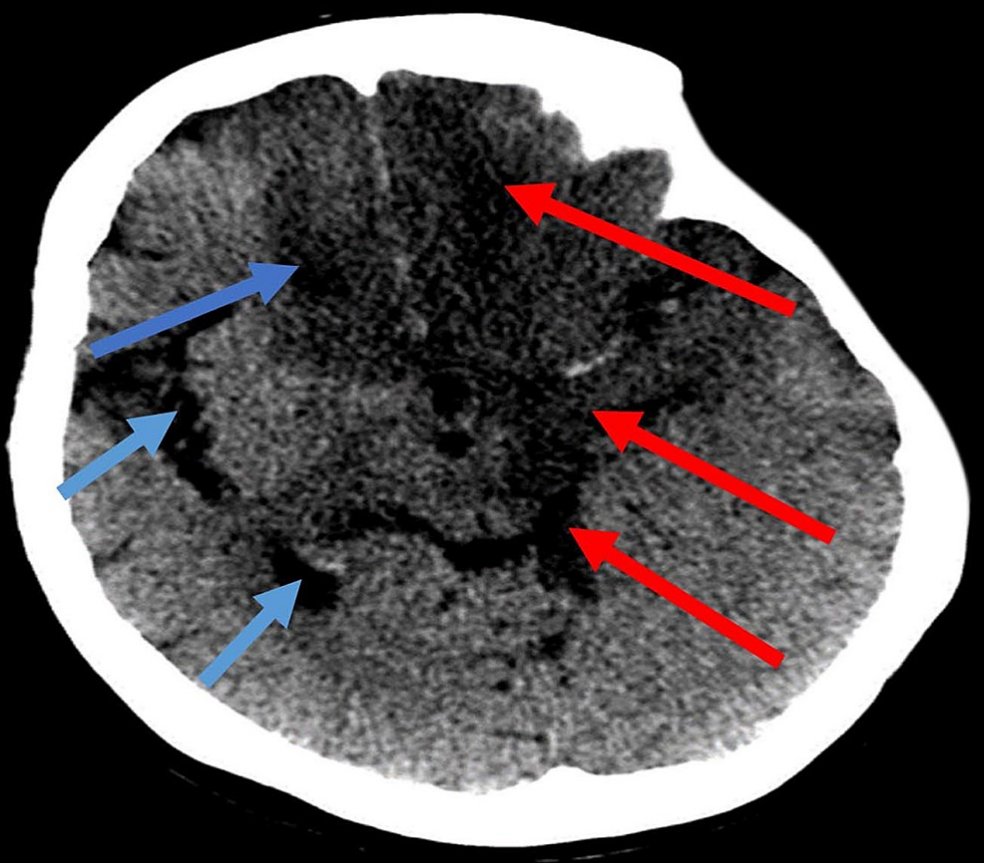
Diffuse anterior, middle and posterior cerebral arterial thrombosis Large ischemia involving the right cerebral hemisphere(red arrows) and left cerebral hemisphere (blue arrows) with midline shift, causing an uncal herniation

Patient was conscious with normal vital signs on admission and CT brain was unremarkable (Figure 1). Her chest radiograph showed bilateral infiltration with extensive peripheral consolidations (Figure 2). Laboratory analysis showed markedly elevated D-dimer, fibrinogen and C reactive protein (CRP). Other laboratory values are presented in Table 1. On day 2 after admission, the patient deteriorated and became unconscious and developed acute renal shutdown. She was started on extensive medical therapy and serial CT angiography. Hematologist was consulted and the patient was shifted from prophylaxis enoxaparin into therapeutic enoxaparin 30 mg IV bolus dose followed 8hr later with maintenance dose enoxaparin 60 mg SC q12hr and monitored with the PTT and fibrinogen level. CT head was obtained on day 2 post admission demonstrating bilateral frontal lobe ischemia (Figure 3). While in hospital, she became increasingly hypoxic and required intubation and mechanical ventilation. Serial CT brain showed diffuse ischemic insult involving left cerebral hemisphere, right frontal lobe and right cerebellum (Figure 4). Her mental function continued to deteriorate with extensive involvement of anterior, middle and posterior cerebral artery complicated with brain edema (Figure 5). The patient remained unresponsive and showed an abnormal breathing pattern on mechanical ventilation. The patient died on day 4 after admission due to severe post-ischemic brain edema, raised intracranial pressure (ICP) and uncal herniation.

**Table 1:** Laboratory results

Measure		Value	Reference Range
White blood cell count/μl		18.8	4-11
Hemoglobin, g/dL		16.5	12-15
Platelet count, X10^3^/L	On admission	230	150-450
	1st day	110	
	2nd day	375	
BUN		30.8	9.8-20.1
Creatinine, mg/dl		1.9	0.6-1.1
Lactic acid, mg/dl		2.9	0.5-2.2
LDH, U/L		483	125-243
Feritin, ng/ml		1077	2.4-4.6
Erythrocyte sedimentation rate (ESR), mm/hr		35	0-15
C-reactive protein, mg/dL		7.8	Less 1
Fibrinogen, g/l		7	2-4
D-dimer, mg/L		2	0-0.5
Prothrombin time (PT)/Second		15.7	11.5-15
Partial thromboplastin time (PTT)/ Second		29.5	25-40
International normalized ratio (INR)		1.2	0.9-1.1

## Discussion

Thrombus formation and pathogenesis of coagulopathy in COVID-19 are incompletely understood. Endothelial injury, microvascular inflammation and/or coagulapathy play a central role in the pathogenesis of acute respiratory distress syndrome and organ failure in patients with severe COVID-19 [6,7]. Studies have shown increased inflammatory markers such as interleukin (IL)-6 and other acute phase reactants and markers of complement activation in the circulation of individuals hospitalized with COVID-19 than in controls [8,9].

The patient in this case report developed a hypercoagulable state with an elevated D-dimer, which could be the main explanation for her extensive arterial thrombosis. Indicators of hypercoagulable state such as hyperviscosity, elevated factor VIII and elevated fibrinogen have been proposed in patients with severe COVID-19 [10,11]. Variable observations existed with respect to the pro-coagulant profile of patients with acute COVID-19 in ICU setting. Two previous studies have reported similar findings consistent with a hypercoagulable state, including very high D-dimers [10,12]. Elevated D-dimer levels were increasingly inversely correlated with overall survival and this were recently reported in Chinese cohort studies [13].

The elevated D-dimer in this patient was treated by boosting the heparin dose. The patient was escalated from prophylaxis enoxaparin 30 mg SC q12hr into therapeutic enoxaparin 30 mg IV bolus dosed followed by maintenance enoxaparin 60 mg SC q12hr. A very recent study showed that prophylaxis anticoagulation was associated with a reduced mortality in more severe COVID-19 patients [14]. This practice was implemented in many institutions [15-17] and was supported by the current guidelines from the leading societies in the field of coagulopathy [18-20]. Nevertheless, in an Italian study of COVID-19 and haemostasis, Mariietta et al. didn’t recommended therapeutic heparin as a standard treatment for all COVID-19 patients and highlighted the importance of taking into consideration the appropriate timing of start of treatment, the type and dosage of drug, and the presence of comorbidities beforehand [21].

The patient in this case report developed an acute renal shutdown, which was reported in literature, and was also related to the elevated D dimer and the high level of inflammatory markers. A complete lack of clot lysis referred to as "fibrinolysis shutdown" was observed on 44 ICU patients and was associated with a high rate of kidney failure and thromboembolic events [22].

The level of the platelet count varies according to the phase of presentation. The patient in this report showed normal platelet levels initially; then the platelet count decreased and when the condition of the patient deteriorated, the platelet count increased. In previous studies, patients with COVID-19 were observed to have higher platelet counts than patients with other coronavirus infections [23]. A study from Ireland reported similar findings to those of high D-dimer and fibrinogen and normal platelet counts and clotting times in an ICU setting [13]. Early case series of patients from Wuhan, China, postulated that low platelet and prolongation of the PT and a PTT were more marked in severe COVID-19 in ICU setting [24-26]. The variation could be explained by delays in patient presentation and/or anticoagulant used for treatment.

The presence of comorbidities increase the chance for arterial thrombosis. The patient in this report had hypertension, which could place her at high risk of thrombus formation. Previous studies support this finding. Reports also showed an increased risk of thrombosis in patients with a history of cardiovascular disease and stroke, as well as hypertension and diabetes [27]. Surprisingly, patients may develop extensive thrombosis even if they have no such medical comorbidities [5].

In this case the patient received prophylactic anticoagulation within the first 24 hours of admission and she developed massive arterial thrombosis while on full dose anticoagulation. The level of D- dimer, and other coagulant and inflammatory markers could limit the benefit of anticoagulation in severe COVID-19 cases and this is why the outcome of patients are different. Prophylactic anticoagulation was found to be associated with improved in-hospital survival in patients with mechanical ventilation [17]. Anticoagulation with low molecular weighted heparin (LMWH) was found to be associated with better outcomes in severely affected cases [1]. The type and dose of the anticoagulant used and early use of anti-coagulant could alter the prognosis of massive arterial thrombosis.

The patient developed massive hemispheric infarction and continued to deteriorate despite aggressive conservative care. Surgical decompression was suggested by the neurosurgeon, but unfortunately the patient died before that. The question raised is whether surgical decompression could improve survival in severe COVID-19 setting? More qualified and large studies are needed to answer this question and to improve the outcome of anti-coagulant use in massive arterial thrombosis

## Conclusions

Massive arterial thrombosis is not uncommon in severe COVID-19 infection. Extensiveness of arterial thromboembolism related to COVID-19 should be evaluated in larger studies in order to estimate the prevalence of this serious complication. All physicians should be aware about severe arterial thrombotic complications as such complications can take place despite the use of antiplatelet or anticoagulant therapy. Decompressive surgery may be required in severe COVID-19 cases complicated with massive hemispheric infarction.
